# Government Intervention, Structural Transformation, and Carbon Emissions: Evidence from China

**DOI:** 10.3390/ijerph20021343

**Published:** 2023-01-11

**Authors:** Shuhua Zhang, Jian Li, Bao Jiang, Tianmiao Guo

**Affiliations:** 1 School of Economics, Ocean University of China, Qingdao 266100, China; 2 Institute of Marine Development, Ocean University of China, Qingdao 266100, China

**Keywords:** government intervention, structural transformation, carbon emissions

## Abstract

Government intervention and structural transformation play an important role in both the economy and carbon emissions. Based on provincial panel data from China from 2003 to 2020, this paper employs econometric models to investigate the impact of government intervention and structural transformation on carbon emissions. In particular, structural transformation is divided into two indicators: The rationalization of the industrial structure and the upgrading of the industrial structure. According to the research findings, government intervention has significantly promoted carbon emissions and structural transformation has had dual effects on carbon emissions; meanwhile, the rationalization of the industrial structure has significantly increased carbon emissions, while the upgrading of the industrial structure has slowed down carbon emissions, with these findings passing the corresponding robustness test. The relationship between government intervention, structural transformation, and carbon emissions varies significantly over time and across regions. Further investigations revealed that government intervention and structural transformation have a significant impact on carbon emissions in various panel quantiles. Finally, the paper makes policy recommendations in order to provide empirical support for promoting China’s high-quality economic development and achieving the “double carbon” goal.

## 1. Introduction

The global economy has undergone rapid expansion since the Industrial Revolution. However, rapid economic development is frequently accompanied by increases in energy consumption, with the corresponding increase in carbon emissions causing side effects that include the greenhouse effect and other environmental problems, thus constituting an impediment to the sustainable development of the global ecosystem and human society. Therefore, methods for reducing carbon emissions and the development of a low-carbon economy have increasingly become the focus of international attention [1,2]. As the world’s largest developing country, China has made remarkable achievements from an economic perspective, but it has long relied on a crude economic growth model characterized by high investment and high energy consumption, with energy consumption outstripping economic growth, resulting in more serious environmental problems such as ecological damage and high carbon emissions [3,4]. According to the former British Petroleum (BP; 2021) [5], China’s total carbon emissions were 1418.5 million tons in 1978. In 2006, China surpassed the United States as the world’s largest carbon emitter. In 2020, China’s carbon emissions reached 9.899 billion tons, accounting for about 30.7% of the global total, ranking first in total global carbon emissions. As an emerging economy with the highest global carbon emissions, China must play a crucial role in the process of global carbon reduction and climate governance. In recent years, effective measures and actions have been taken to reduce carbon emissions, demonstrating the world’s firm determination to reduce carbon emissions [6]. In 2011, the first carbon emissions trading system was established and is the best way to promote both low carbon emissions and economic development [7] The necessity of promoting a sound economic structure that facilitates green, low-carbon, and circular development was presented at the 19th National Congress of the Communist Party of China in 2017. In 2020, General Secretary Xi Jinping delivered a great speech at the 75th Session of the United Nations General Assembly, stating that China will strive to reach peak carbon dioxide emissions before 2030 and will achieve carbon neutrality by 2060, indicating an important strategic deployment by China aimed at creating a communal life for humans and nature. The formulation of carbon emission quota allocations in 2021 is expected to become an important policy tool for achieving China’s “double carbon” goal.

The academic community has also paid a great deal of attention to methods for reducing carbon emissions and achieving the “double carbon” goal. Previous research has demonstrated that policy factors such as carbon emissions trading [7,8], the allocation of carbon emissions quotas [9], and operational frameworks and mechanisms [10] cannot be overlooked in the pursuit of reducing carbon emissions. They play an important role in mitigating carbon emissions. Furthermore, related research has shown that structural transformation is the main driving force and an important mechanism for reducing carbon emissions in the process of China’s development, which is of great practical significance in achieving China’s “dual carbon” goal [11,12,13]. Indeed, structural transformation can promote reasonable industrial structure, strengthen coordination and cooperation among industries, optimize resource allocation, promote industrial transformation and structural upgrade, eliminate backward industries, improve overall production efficiency, and effectively reduce carbon emissions [14]. In summary, government participation and structural transformation are indispensable in the process of reducing carbon emissions. Therefore, the manner in which their coordinated development affects carbon emissions must be further investigated. In light of this, in this paper, a thorough investigation and analysis of the impact of government intervention, structural transformation, and coordinated development on carbon emissions, along with their mechanisms of action, is carried out, and the conclusions are crucial for promoting low-carbon and green economic development and achieving the goals of “carbon peaking” and “carbon neutralization.”

The main contributions of this paper are as follows: we seek to illuminate the complexities of industrial transformation; we investigate not only the rationalization of the industrial structure, but also its upgrade, and integrate government intervention, structural transformation, and carbon emissions into a unified analytical framework to investigate the impact of government intervention, structural transformation, and their coordinated development on carbon emissions; we consider the heterogeneity of the eastern, central, and western regions arising from regional advantages with respect to resource endowment, economic foundations, industrial foundations, etc., and investigate the relationship between government intervention, structural transformation, and carbon emissions in a way that is more in line with economic development and practical needs.

The remainder of this paper is structured as follows. Section 2 presents a review of the relevant literature. Section 3 discusses the construction of the econometric model, selection of variables, and describes the data employed. Section 4 presents an analysis of the empirical findings. Section 5 presents our findings and policy recommendations.

## 2. Literature Review

Carbon emissions have become a focus of academic research in recent years. On the basis of a review of the literature, it was found that many scholars have analyzed and predicted the logical mechanisms governing the impact of government intervention and structural transformation on carbon emissions. In this paper, on the basis of a review of the existing literature, we will focus on the following two topics.

### 2.1. Impact of Government Intervention on Carbon Emissions

The government plays an important role not only in the process of economic growth but also in regional environmental governance and the reduction of carbon emissions. Previous research has shown that the relationship between the government and carbon emissions is more complex and diverse than simple promotional or inhibitory effects. To begin with, government intervention has a positive impact on carbon emissions. Competition among local governments is an important factor to consider in the context of the Chinese style of decentralization. Local governments make “self-interested” and “short-term” decisions in response to the necessities of local competition and performance appraisals [15]. Long-term reliance on models characterized by high energy consumption and extensive economic development in order to accelerate economic development results in increased consumption of energy and other resources, resulting in higher carbon emissions [16]. Xia et al. believed that the short-sighted economic catch-up behavior of local governments has exacerbated environmental damage and increased carbon emissions [17]. According to the research of Zhang et al. [18], local governments have pursued the strategy of a “race to the bottom”. Environmental regulatory standards and access thresholds were kept low, resulting in a large number of high-capacity, polluting industries pouring in, exacerbating local pollution and carbon emissions. Second, government intervention has resulted in a significant reduction in carbon emissions. With the diversification of performance appraisals, the weighting given to environmental performance has increased. Local governments have taken appropriate measures to improve regional environmental protection and have played an important role in reducing carbon emissions [19,20,21]. Finally, the relationship between government intervention and carbon emissions is complex and nonlinear. Wang et al. discovered a nonlinear U-shaped relationship between government intervention and carbon emissions [22], and Xia et al. discovered a three-stage nonlinear dynamic relationship between government intervention and carbon emissions [23]. In summary, the mechanism of action of government intervention in carbon emissions is complex, but they are inextricably linked.

### 2.2. Impact of Structural Transformation on Carbon Emissions

The impact of structural change on carbon emissions has sparked widespread interest among academics. According to the relevant literature, the relationship between structural transformation and carbon emissions can be observed in the following ways: First, at the level of industrial structure rationalization, many studies have confirmed the hypothesis that industrial structure rationalization is beneficial to reducing carbon emissions. Li et al. discovered that the rationalization of the industrial structure leads to improvements in industry coordination, the optimization of allocation among industries, improvements in resource use, and reductions in carbon emissions [24]. On the basis of empirical research using econometric models, Lu et al. reported that the rationalization of industrial structures has a significant inhibitory effect on carbon emissions [25]. Second, with respect to improvements in the industrial structure, many academics believe that encouraging the development of advanced industrial structures is an important way of studying and developing regions with the aim of meeting emissions reduction targets [26,27]. Third, with respect to industrial transformation, the existing literature has primarily addressed the industrial structure from the standpoint of either the rationalization or the upgrade of the industrial structure individually. In fact, structural transformation consists of two primary components: the rationalization and the upgrade of the industrial structure [28]. Structural transformation is an effective means of reducing future carbon emissions and should be a topic of focus [29,30]. Some related research has shown that these two indexes may exert influence in different ways. Chen et al. reported, on the basis of empirical research, that the rationalization and upgrade of the industrial structure in structural transformation can significantly curb carbon emissions [12,31]. Some scholars believe that the rationalization of the industrial structure has a positive relationship with carbon emissions, while upgrading the industrial structure is conducive to reducing carbon emissions [28,32]. This paper analyzes structural transformation from the perspectives of the rationalization or the upgrade of the industrial structure in order to comprehensively explore in depth the impact of structural transformation on carbon emissions.

Despite the fact that there are numerous beneficial discussions on the impact of government intervention on carbon emissions and the relationship between structural transformation and carbon emissions in the existing literature, laying a solid foundation for the current research, the existing research has only looked at carbon emissions from a single perspective, that is, with respect to either government intervention or structural change. The conclusions of such research are biased and require additional research and analysis. In light of this, in this study, government intervention, structural transformation, and carbon emissions are systematically integrated into a single analytical framework, and structural transformation is divided into two parts: industrial structure rationalization and industrial structure upgrade. We will investigate the internal mechanisms and impact mechanisms of these two components on carbon emissions in order to provide a reference for the decision-making process with the aim of achieving the high-quality development of China’s economy under new development patterns while also providing a path for the realization of the “dual carbon” goal. On the basis of the theoretical analyses described above, this paper proposes Hypotheses 1–3:

**Hypothesis** **1** **(H1).** *Government intervention may exacerbate carbon emissions*.

**Hypothesis** **2** **(H2).** *Structural transformation has a dual effect on carbon emissions*.

**Hypothesis** **3** **(H3).** *The interaction between government intervention and structural transformation has a dual effect on carbon emissions*.

## 3. Model and Data

### 3.1. Model Specification

This paper examines the relationship between government intervention, structural transformation, and carbon emissions using data from China’s provincial panels from 2003 to 2020, on the basis of the following benchmark measurement model:

(1) $$lnAC_{it}=\alpha_{0}+\beta lnGI_{it}+\gamma lnST_{it}+\delta lnGI_{it}*ST_{it}+\varphi Controls_{it}+\varepsilon_{it}$$

where *i* represents provinces and cities in China, *t* represents the year, and *AC* represents carbon dioxide per capita (kg per capita); *GI* means government intervention; *ST* refers to structural transformation (i.e., RIS for the rationalization of the industrial structure and UIS for the upgrading of the industrial structure); *Controls* represents a series of control variables, including openness, foreign direct investment, human capital, environmental regulation, and the urbanization level; Variate $\alpha_{0}$ is a constant term, β,  γ, and δ are the regression coefficients of each explanatory variable, *Controls* is the estimated parameter of the control variable, and $\varepsilon_{it}$ represents the random error term.

### 3.2. Variable Definitions

#### 3.2.1. Explained Variable

##### Carbon Emissions Per Capita (AC)

With the acceleration of global industrialization and urbanization, energy consumption, particularly the consumption of traditional fossil fuels, is rising each year. As a result, total carbon dioxide emissions will continue to rise. In combination with China’s actual carbon emissions, the Intergovernmental Panel on Climate Change (IPCC) proposed a reference method in 2006 for estimating total carbon emissions by determining the consumption of eight fossil fuels closely related to carbon emissions: diesel, coal, coke, kerosene, gasoline, fuel oil, natural gas, and crude oil. As shown in Formulas (2) and (3), the per capita carbon emissions of each region are the ratio of the total regional carbon emissions to the total regional population.

(2) $$CO_{2}=\sum_{i=1}^{H}E_{i}$$

(3) $$CEF_{i}=H_{i}\times CH_{i}\times COR_{i}\times CE_{i}\times \frac{44}{12}\times 10^{-6}$$

where $E_{i}$ represents the total consumption of fossil energy source *i*; $CEF_{i}$ is the carbon emission coefficient of energy source *i*; *H*, $CH_{i}$, $COR_{i}$, and $CE_{i}$ represent the average low calorific value, carbon content per unit of the calorific value, the carbon oxidation rate, and the final carbon emission coefficient, respectively. See Table 1 for the definitions of other indicators.

#### 3.2.2. Core Explanatory Variables

The core explanatory variables include government intervention (*GI*) and structural transformation (*ST*).

Government intervention (*GI*). Government intervention relates to policies and measures that have played an important role in reducing carbon emissions. With reference to the treatment methods described in [33], the proportion of fiscal expenditure is determined as a percentage of GDP for each region in order to assess the level of regional government intervention.

Structural transformation (*ST*). Structural transformation has two dimensions: static coordination of the industrial structure layout and the dynamic upgrade of the industrial structure efficiency, that is, the coordinated development of industry and the evolution of the industrial structure. In this paper, structural transformation is divided into two aspects on the basis of existing research: the rationalization and upgrading of the industrial structure.

Rationalization of the Industrial Structure (RIS). The rationalization of the industrial structure refers to the degree of coordination between industries and the effective use rate of resource elements among industries. This paper refers to the research of Frank (2014) [19], in which the Thiel index was employed to assess the level of rationalization of the regional industrial structure. The calculation formula is as follows:

(4) $$RIS=\sum_{k}^{n}\left(Y_{k}/Y\right)ln\left(\frac{Y_{k}/L_{k}}{Y/L}\right),\;n=1,2,3$$

where *RIS* represents the Thiel index, namely, the level of the industrial structure rationalization, *Y* represents the output value, *L* represents the number of employees, *k* represents the number of industrial sectors, *n* represents the number of industrial sectors, and Y/L represents productivity. The Theil index reflects the balance of the regional economic structure. If the economy is in equilibrium, RIS = 0, meaning that the industrial structure is reasonable, while RIS ≠ 0 indicates an unreasonable industrial structure. Greater deviations in the value from 0 indicate more unreasonable industrial structures.

Upgrading of the industrial structure (*UIS*). The upgrading of the industrial structure refers to the transition of the industrial structure from labor-intensive industries to capital-intensive industries, and subsequently to knowledge- and technology-intensive industries and traditional industries. According to previous research [34], the output values for the tertiary industry and secondary industry can be used to measure the upgrading of the industrial structure. The higher the value of this indicator, the higher the degree to which the industrial structure has been upgraded.

#### 3.2.3. Control Variables

On the basis of previous research, in this paper, we focus primarily on the following control variables: Opening (*OU*). The total imports and exports of provinces and regions as a proportion of GDP are used in this paper to measure regional openness [35]. Foreign direct investment (*FDI*). The level of regional FDI using FDI as a proportion of GDP in each province [36]. Environmental Regulation (*ER*). Investment in environmental pollution control as a proportion of GDP is used to measure the level of environmental regulation in each province [37]. Human capital (*HR*). The number of years of education per capita in each province is used to measure human capital. Urbanization Level (*UR*). Urban population as a proportion of the total population is used to measure the urbanization level in each province [31].

### 3.3. Data Resources and Data Description

Considering the availability, validity, and continuity of data, as well as the statistical time. This paper is based on a panel of 30 provinces from 2003–2020 in China (excluding Hong Kong, Macao, Taiwan, and Tibet) to empirically determine the relationship between government intervention, structural transformation, and carbon emissions. The data were obtained from the China Environmental Statistical Yearbook, the Wind Database, the China Statistical Yearbook, the China Industrial Statistical Yearbook, the China Energy Statistical Yearbook, the China Population and Employment Statistical Yearbook, the China Labor Statistical Yearbook, the China Science and Technology Statistical Yearbook, and the Statistical Yearbooks of the Provinces and Cities. The data were processed as necessary in order to address problems such as repeated data samples, measurement errors, and missing index data in the databases. To reduce the bias of the model, all variables were processed logarithmically. Descriptive statistics are provided for each variable in Table 2.

## 4. Empirical Analysis

### 4.1. Benchmark Regression Analysis

To analyze the effects of the variables in detail, the results were further explored on the basis of FE models. The benchmark regression results for the impact of government intervention, structural transformation, and the interaction between government intervention and the rationalization of the industrial structure on carbon emissions are reported in Table 3. Table 3 (1), (2), and (3), respectively, show the impact of government intervention, rationalization of industrial structure, and upgrading of industrial structure on carbon emissions. Columns (4) and (5) in the table show the impact of the interaction of government intervention and structural transformation on carbon emissions.

First, the impact of government intervention on carbon emissions was examined. The regression coefficient for government intervention was positive, that is, government intervention has had a significant promotional effect on carbon emissions. Local governments have exhibited short-sighted behaviors, such as unilaterally pursuing GDP at the expense of the environment over a long period of time [31], and have a high degree of dependence on traditional industries characterized by high resource consumption, which has exacerbated regional carbon emissions to a certain extent.

Second, the impact of structural transformation (the rationalization and upgrade of the industrial structure) on carbon emissions, was evaluated. The empirical results obtained for the effect of structural transformation on carbon emissions are consistent with those reported by Feng et al. [28]. The rationalization of the industrial structure has resulted in a significant increase in carbon emissions. From the perspective of China’s actual development, the development of regional industries and industrial structure has been chaotic. The resulting “distortion” effect has led to low efficiency in the allocation of resource elements among industries, significantly increasing carbon emissions. The upgrade of the industrial structure demonstrates an obvious inhibitory effect on carbon emissions. The “upgrading effect” of upgrading the industrial structure effectively promotes the transition from industries characterized by high energy consumption, high emissions, and low returns toward green industries characterized by low energy consumption, low emissions, and high returns improving the coupling between the input and output of production factors, and leading to reductions in carbon emissions.

Third, with respect to the impact of the interaction between government intervention and the rationalization of the industrial structure on carbon emissions, it can be seen that the interaction term indicates a significant intensification of carbon emissions. This is because promotion incentives centered on GDP result in local governments scrambling to pursue economic performance, adopting a “race to the bottom” strategy [38], and unilaterally developing high-value, high-quality-taxation, high-pollution, and high-consumption industries, resulting in the development of unreasonable industrial structures, low use rates of resources and energy, and the exacerbation of regional carbon emissions. The coefficient for the effect of government intervention and the industrial structure upgrade is significantly negative. It can be seen that the interaction term demonstrates a significant inhibition of carbon emissions, indicating that local governments have fully tapped their “structural dividends” to achieve a transformation in fiscal expenditure and innovation with respect to polluting enterprises. Industrial policies and support for technological transformation support comprehensively improve the production efficiency of industrial factors and promote the transformation and upgrade of regional industrial structures in the direction of green and low-carbon industries, thus weakening regional carbon emissions.

Finally, among the control variables, the coefficient for the impact of FDI on carbon emissions is positive, but not significant. It can be seen that FDI still aggravates carbon emissions as a result of the lower environmental standards of developing countries. High-energy-consumption and high-pollution enterprises have resulted in a “pollution haven” effect and “carbon leakage” problems, significantly increasing carbon emissions. Human capital is conducive to reducing carbon emissions, indicating that improvements in the level of human capital are able to effectively improve the efficiency of production technology, improve resource and energy use efficiency, and slow down carbon emissions. Environmental regulation is positive and significant, indicating that current environmental regulations have not played a role in reducing carbon emissions. This may be due to the insufficiency of local government environmental regulations, low environmental protection thresholds for industry access, and the presence of many pollution-intensive industries in the region, resulting in high carbon emissions. Urbanization plays a role in the promotion of carbon emissions. In the process of promoting urbanization in China, the high demand for energy and the rapid increase in energy consumption have led to increased carbon emissions.

### 4.2. Endogenetic Test

To solve the problems stemming from endogeneity and estimation error that may occur in static panel models, in this paper, the GMM model dynamic panel system was used for estimation, and the regression results are shown in Table 4. The *p*-value obtained for the Sargan test was greater than 0.1, indicating that there was no problem related to the overidentification of the tool variables. The results of the AR (1) and AR (2) tests indicated that the random error term exhibited first-order autocorrelation, but there was no second-order autocorrelation, which means that the problem of the model’s endogeneity was solved.

### 4.3. Robustness Test

To avoid the “pseudo regression” problem in the quantitative empirical results regarding the effects of government intervention and structural transformation on carbon emissions and to further ensure the robustness and credibility of the benchmarking regression results, in this paper, two strategies were employed to test robustness: replacement of the explained variable and a reduction of the time sample.

#### 4.3.1. Replacement of the Core Explanatory Variable

To prevent bias in the research conclusions due to excessive reliance on a single measurement indicator, total carbon emissions (TC) were used as an alternative indicator for per capita carbon emissions (AC) for the purposes of re-regression analysis. It can be seen from Table 5 that the regression results for the robustness of the effect of government intervention and structural transformation on carbon emissions are basically consistent with the baseline regression results, indicating the reliability of the baseline regression results.

#### 4.3.2. Reduction in the Time Scale

To further test the robustness of the benchmark empirical results reported in this paper, the data from 2004 to 2019 were selected to test for robustness, and the parameters of the benchmark regression model were re-estimated. The regression results for the robustness test are presented in Table 6. It can be observed that the direction and significance of the main explanatory variables with respect to carbon emissions are highly consistent with the baseline regression results, indicating that the conclusions presented in this paper are relatively robust.

### 4.4. Heterogeneity Analysis

To further explore whether there were differences in the impact of government intervention and structural transformation on carbon emissions, we drew on conventional practices to analyze heterogeneity in terms of time and space.

#### 4.4.1. Time Heterogeneity

In 2011, the Chinese government issued the Notice of Pilot Work on Carbon Emission Rights Trading, which has had a great impact on China’s carbon emissions. In view of this, 2011 was taken as a time node in this paper to explore the time difference between government intervention, structural transformation, and carbon emissions. The regression estimation results are shown in Table 7.

First, the impact of government intervention on carbon emissions exhibited a positive correlation in 2003–2011 and a negative correlation in 2012–2020. The main reason for this may be that, against the backdrop of economic performance being the main factor influencing the promotion of local officials, local government officials make “self-interested” and “short-term” decisions, focusing on the development of resource-intensive and labor-intensive industries characterized by high energy consumption and the production of serious waste, leading to increased regional carbon emissions. Since 2011, China’s economic development has gradually transformed, moving into a stage of high-quality economic development. The central government attaches great importance to environmental protection and has implemented a series of measures such as the carbon property rights trading policy. The “weighting” of environmental governance in government officials’ performance appraisals has increased significantly. The government has changed from the “race to the bottom” model employed in the past to gradually strengthening environmental protection and supervision, effectively changing the structure of energy consumption, promoting the use of clean energy, improving the efficiency of the consumption of resources and energy, and, to a certain extent, reducing carbon emissions.

Second, in terms of the impact of structural transformation (rationalization and upgrade of the industrial structure) on carbon emissions, it can be seen that from 2003 to 2011, the effect of the industrial structure rationalization on carbon emissions was significantly greater than that from 2012 to 2020. In addition, the effect of the industrial structure rationalization on carbon emissions gradually weakened. This indicates that, with the adjustment of the industrial structure, there has been a continuous improvement in the degree of regional industrial coordination, resource elements have been able to flow freely and rationally among industries, and the increase in carbon emissions has gradually reduced. During the 2003–2011 period, the upgrading of the industrial structure significantly alleviated carbon emissions, while during the 2012–2020 period, carbon emissions were suppressed, although the coefficient was not significant, and there was still a promotional effect on the reduction of carbon emissions. This shows that upgrading the industrial structure is an effective measure for reducing carbon emissions, which is in line with the expected results. Upgrading the industrial structure can effectively eliminate backward capacity, promote industrial development to shift towards high added-value and high-technology industries, reduce the prevalence of high energy-consumption and high-pollution industries, promote the development of green industry, and effectively reduce carbon emissions.

Finally, when examining the impact of interactions on carbon emissions, it was observed that the interaction between government intervention and the rationalization of the industrial structure in 2003–2011 contributed significantly more to carbon emissions compared with 2012–2020. This indicates that the Chinese government needs to formulate differentiated and precise industrial policies that take into consideration the characteristics of local industrial development and bring into play the anticipated carbon emission reduction effect of the industrial structure rationalization. For both 2003–2011 and 2012–2020, the interaction coefficient between government intervention and the optimization of the structure was negative, but not significant, indicating an overall inhibitory effect on carbon emissions. It is known that with high-quality economic development and green upgrade, the industrial structure and carbon emissions gradually become important assessment indicators for the performance of local government. Local governments have given full play to the advantages of macro-control by mechanisms such as financial subsidies, tax exemptions, land preferences, and other means, in order to improve the level of the industrial structure, promote the use of new energy-saving and environmental protection technologies, reduce regional carbon emissions to bring them more into line with the regional low-carbon economic development model, and build a beautiful home with green mountains and water.

#### 4.4.2. Regional Heterogeneity

Different regions in China have different degrees of economic development and levels of industrial structure, different levels of resource consumption, and different levels of carbon emissions. Therefore, in different regions, the impact of government intervention and structural transformation on carbon emissions may also be different. To analyze this heterogeneity, the whole sample was divided into eastern and western regions in this paper in order to explore regional differences in the impact of government intervention and structural transformation on carbon emissions, as estimated in Table 8.

First, the regression coefficient of government intervention on carbon emissions, regardless of the eastern, central, or western regions, indicated a significant increase in carbon emissions, with little difference among regions. It is a reality in China that, in the pursuit of political interests and economic development, local governments have not completely rid themselves of the extensive development mode characterized by high energy consumption, high pollution, high emissions, and increased consumption of resources and energy, which is bound to increase carbon emissions.

Furthermore, when considering the impact of structural transformation (industrial structure rationalization and upgrade) on carbon emissions, it can be seen that the rationalization of the industrial structure in the eastern region has an obvious promotional effect on regional carbon emissions, but this is not the case in the central and western regions. For the economically developed eastern region, industrial development occurred earlier, but there are more low-end resource-intensive industries, in addition to unbalanced industrial development, poor allocation of production resources, and serious resource wastage, exacerbating carbon emissions. However, the central and western regions are severely constrained by their economic backwardness, lack of resources, and other factors; the industrial foundations are weak, and the promotional effect on carbon emissions is small. Industrial structure upgrade in the eastern region significantly increased regional carbon emissions, indicating that the economically developed eastern region is characterized by a large space and market for industrial development, the rapid development of technology-intensive and knowledge-intensive industries, low dependence on resource-intensive industries with high energy consumption, and considerable development of low-carbon economies, which is conducive to the suppression of carbon emissions. The impact of upgrading the industrial structure on carbon emissions in the Midwest region was negative and not significant. It is possible that the economic development in the Midwest region is backward, the infrastructure is not perfect, the accumulation of capital and technology has been relatively scarce as a result of industrial transfer from the eastern region, the upgrade of the industrial structure is limited, and its inhibitory effect on carbon emissions has not yet been shown.

Finally, with respect to the regression coefficients of the effects of the interactions of these two factors on carbon emissions, the interaction of government intervention and the rationalization of the industrial structure in the eastern region has a significantly higher positive effect on carbon emissions than in the central and western regions. This is because, under the appraisal system based on economic performance, the local governments in the eastern region, which have their own economic advantages, increase their investment in high-capacity, high energy-consumption, and high-pollution industries, excessively interfere with the allocation of production resources among industries, resulting in unbalanced industrial development and a serious waste of resources and energy. In the central and western regions, due to their weak economic strength, the relatively lower level of investment in high energy-consumption industries, and the low degree of intervention in industrial rationalization, the promotion of regional carbon emissions is small. The interaction between local government intervention and the upgrading of the industrial structure in the eastern region has a significant inhibitory effect on regional carbon emissions, while it has a significant promotional effect in the central and western regions. This is because, on the one hand, the eastern regions, which are characterized by better economic development and better infrastructure, are able to provide conditions more conducive to the formulation and implementation of industrial policies by governments and the effective promotion of carbon emissions reduction policies, thus promoting the development of low-carbon industries and actively curbing carbon emissions. On the other hand, the eastern region has a high level of economic development. The government has invested more funds in the promotion of technological innovation, improving the overall production efficiency of industry, promoting the upgrade and transformation of traditional industries in order to improve value-added technology, promoting the use of clean energy and other environmental protection technologies, and effectively promoting and curbing carbon emissions. However, the economy in the central and western regions is backward, and the foundations for industrial development are weak. In the process of promoting the transformation and upgrade of traditional industries and eliminating backward production capacity, it is difficult for the effect of energy conservation and emission reduction caused by the government to offset the growth in carbon emissions caused by high-pollution and high energy-consumption industries, thus intensifying regional carbon emissions.

### 4.5. Further Analysis

**Figure 1:**
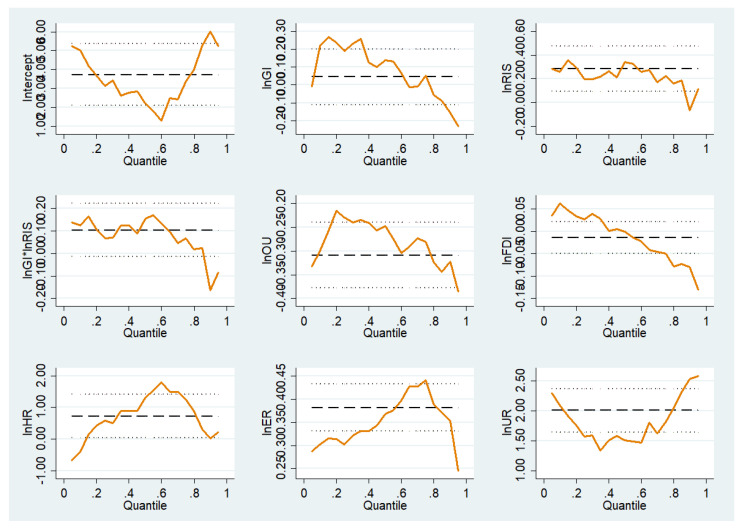
Variation of quantile regression coefficients for each factor.

**Figure 2:**
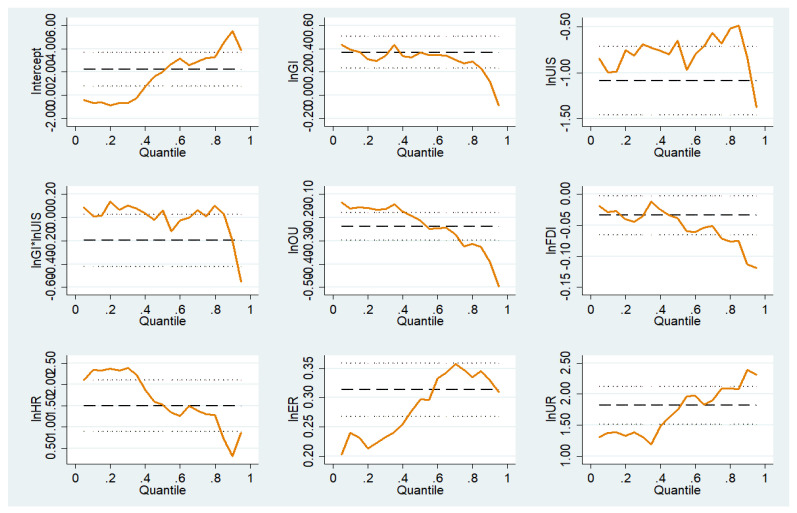
Variation in the quantile regression coefficient of each factor.

In further exploring the internal mechanisms at play among government intervention, structural transformation, and carbon emissions, we took into account the data peak and fat tail, heteroscedasticity, and other issues that can cause deviations in the data to ensure that the measured and estimated results of carbon emissions as a result of government intervention and structural transformation would not be affected by extreme values. Five representative quantiles—10%, 25%, 50%, 75%, and 90%—were selected for the purposes of description, and the bootstrap method was used for the panel data to estimate the standard error of the coefficients. The regression results are shown in Table 9 and Table 10; Figure 1 and Figure 2.

First, by examining the impact coefficient of government intervention on carbon emissions, it can be observed that the intensity of government intervention in the promotion of carbon emissions is characterized by dynamic changes. Specifically, on the basis of the distribution of conditions from the low-end quantile to the high-end quantile, it can be observed that the role of government intervention in the promotion of carbon emissions gradually weakens or even disappears. It can be seen that the inhibitory effect of government intervention in high-quantile provinces is negligible, with a greater promotional effect obtained for government intervention in low-quantile provinces. To pursue economic development, local governments have long relied on an economic growth model, which has come at the cost of the consumption of resources and the occurrence of environmental damage, which has significantly increased carbon emissions.

Second, with respect to the coefficient of structural transformation (rationalization and upgrading of the industrial structure) for carbon emissions, it can be seen that the coefficient of the industrial structure rationalization gradually decreases from the low quantile to the high quantile and becomes negative, indicating that the rationalization of the industrial structure has the effect of promoting and then inhibiting carbon emissions. As the industrial base of low-quantile provinces is relatively weak, the increase in energy consumption caused by low-end resource-intensive industries has a greater impact on low carbon-emission regions. High-quantile provinces have a good foundation for industrial development and reasonable resource allocation, which can effectively improve the efficiency of resource and energy use, resulting in the unit value of carbon emissions being lower than that in low-quantile provinces, having a positive inhibitory effect on carbon emissions. Upgrading the industrial structure has a significant inhibitory effect on carbon emissions, and there is little difference between provinces from different quantiles. This is because, in the process of advancing the development of the industrial structure, accelerating the elimination of backward and high energy-consumption industries, promoting the development of industrial technology innovation, improving the overall production efficiency of industry, and reducing the consumption of resources and energy all have a positive inhibitory effect on carbon emissions.

Finally, by examining the regression coefficient of their interaction terms with carbon emissions, it can be found that the interaction between government intervention and the rationalization of the industrial structure plays a strong role in promoting carbon emissions in low-quantile provinces, while this effect becomes inhibitory in high-quantile provinces. The reason for this is that in low carbon-emission regions, the government intervenes excessively in industrial development and issues unreasonable industrial policies, leading to a relative imbalance in industrial development and blocking the reasonable flow of factor resources, resulting in a low energy use rate and increased carbon emissions. In high carbon-emission areas, the government should intervene properly in the rational development of the industrial structure, improve the allocation of resource elements among industries, and significantly improve the problem of carbon emissions. The interaction between government intervention and the upgrading of the industrial structure has no significant impact on carbon emissions. In reality, in China, regional economic development has gradually changed from an extensive development model to a low carbon-emission, green, high-quality development model. Knowledge-intensive industries dominated by tertiary industries have developed slowly, failing to achieve the expected effects in terms of carbon emissions.

## 5. Conclusions

China is not only the largest emerging economy in the world; it is also the country that produces the highest amount of carbon emissions. The contradiction between carbon emissions and high-quality economic development has become increasingly prominent. In this context, it is of great practical significance to further clarify the logical and operational mechanisms related to government intervention, structural transformation, and carbon emissions with the aim of realizing China’s “double carbon” goals while also achieving high-quality economic development. Accordingly, a framework was developed in this paper that incorporates government intervention and structural transformation, and the relationships among government intervention, structural transformation, and carbon emissions were explored deeply on the basis of the provincial panel data from 2003 to 2020, as well as with empirical testing. On this basis, the following conclusions can be drawn: (1) Government intervention and structural transformation complement each other and jointly promote carbon emission reduction. This conclusion holds true when subjected to multiple robustness tests, which has strong policy implications in terms of the country’s ability to achieve its “dual carbon” goal. (2) The heterogeneity test demonstrated that government intervention and structural transformation had an inhibitory effect on carbon emissions, with the upgrading of the industrial structure being more significant in the eastern region due to its better industrial foundations and more developed economy. (3) Further research found that government intervention, structural transformation, and their interaction terms indicated significant heterogeneity among the different quantiles with respect to carbon emissions.

On the basis of the above findings with respect to policy implications, we present the following recommendations.

(1) To improve the role played by the government with respect to carbon emissions, it is necessary for the central government to abandon “GDP-based” local government performance evaluation mechanisms, promote diversified performance evaluation standards, further improve carbon emission performance, incorporate “green GDP” into the performance evaluation system, improve environmental protections, promote the development of a green and low-carbon economy, and effectively curb the effect of carbon emissions.

(2) Give full play to the advantages of factor endowments and economic development levels in different regions, implement relevant policies accurately and according to local conditions, promote regional structural transformation, promote low-carbon transformation or industrial transfer in industrial sectors characterized by high energy consumption and high emissions, and improve the efficiency of resource and energy allocation, so as to “vacate the cage and change the bird” for the development of low-carbon industries in the region, fully enable the development of low-carbon transformation in the region, and effectively reduce carbon emissions.

(3) Furthermore, give full play to the country’s unique political and institutional advantages with respect to reducing carbon emissions and focus on promoting a carbon emission model that combines government and structural transformation with complementary advantages. Policies should be implemented to accelerate structural transformation, especially with respect to increasing the degree of sophistication in the industrial structure, and to achieve the comprehensive and sustainable development of the green economy.

Nevertheless, this study has some limitations with respect to exploring the relationship between government intervention and structural transformation and carbon emissions, mainly in terms of two aspects. First, this paper was focused on the impact of China’s provincial-level economic growth goals and carbon emissions and lacks an in-depth discussion of China’s urban level, which may result in some deviations. Secondly, in addition to the factors affecting the regional carbon emissions addressed in this study, there are other relevant factors, such as local fiscal decentralization and the upgrading of the industrial structure, that were not taken into account. These shortcomings provide a direction for our future research work.

## Figures and Tables

**Table 1:** Conversion table of the carbon emission coefficient of the major energy sources.

Number	Energy Category	Average Low Calorific Value H_i_ (KJ/KG)	Carbon Emission Factor CH_i_ (TC/TJ)	Carbon Oxidation Rate COR_i_	Carbon Emission Coefficient CEF_i_ (KGCO_2_/KG)
1	diesel oil	42,652	20.2	0.98	3.10
2	coke	28,435	29.5	0.93	2.86
3	coal	20,908	26.4	0.94	1.90
4	kerosene	43,070	19.5	0.98	3.02
5	gasoline	43,070	18.9	0.98	2.93
6	fuel oil	41,816	21.1	0.98	3.17
7	natural gas	38,931	15.3	0.99	2.16
8	crude oil	41,816	20.1	0.98	3.02

**Table 2:** The descriptive statistics of the variables.

Variable Type	Variables	Obs.	Mean	Std.	Min.	Max.
Explained variable	*lnAC*	540	1.992	0.643	0.347	3.956
Explanatory variable	*lnGI*	540	−1.567	0.423	−2.474	−0.277
	*lnRIS*	540	−0.733	0.868	−4.828	0.778
	*lnUIS*	540	0.795	0.383	−0.641	1.667
Control variable	*lnQU*	540	−1.674	0.979	−4.883	0.537
	*lnFDI*	540	5.037	1.626	−1.220	7.495
	*lnHR*	540	2.167	0.117	1.798	2.548
	*lnER*	540	−5.921	0.868	−10.022	−2.271
	*lnUR*	540	−0.658	0.272	−1.394	−0.640

**Table 3:** Estimated results on the basis of the basic model.

Variables	Explained Variable: *lnAC*				
	(1)	(2)	(3)	(4)	(5)
*lnGI*	0.636 ***			0.869 ***	0.689 ***
	(0.065)			(0.065)	(0.060)
*lnRIS*		0.049		0.774 ***	
		(0.034)		(0.084)	
*lnUIS*			−0.367 ***		−0.509 ***
			(0.048)		(0.115)
LnGI*lnRIS				0.428 ***	
				(0.049)	
LnGI*lnUIS					−0.066
					(0.071)
*lnQU*	0.060 **	−0.006	−0.053 *	−0.002	0.003
	(0.029)	(0.031)	(0.030)	(0.027)	(0.027)
*lnFDI*	0.018	0.063 ***	0.055 ***	0.011	0.006
	(0.015)	(0.016)	(0.015)	(0.014)	(0.014)
*lnHR*	−0.835 ***	−0.130	0.255	−0.375 *	−0.320
	(0.228)	(0.254)	(0.237)	(0.223)	(0.218)
*lnER*	0.048 ***	0.059 ***	0.059 ***	0.044 ***	0.046 ***
	(0.013)	(0.014)	(0.013)	(0.012)	(0.012)
*lnUR*	1.034 ***	1.433 ***	1.499 ***	0.926 ***	1.103 ***
	(0.108)	(0.111)	(0.105)	(0.101)	(0.100)
Constant	5.774 ***	3.274 ***	2.443 ***	5.016 ***	4.775 ***
	(0.564)	(0.584)	(0.551)	(0.537)	(0.531)
Observations	540	540	540	540	540
Number of id	30	30	30	30	30
R-squared	0.636	0.568	0.611	0.689	0.692

Note: *p*-values in square brackets, *** *p* < 0.01, ** *p* < 0.05, * *p* < 0.1. The Z value of the system test is shown in parentheses.

**Table 4:** Dynamic panel regression results.

Variables	Explained variable: *lnAC*				
	(1)	(2)	(3)	(4)	(5)
*L.lnAC*	0.680 ***	0.824 ***	0.742 ***	0.568 ***	0.504 ***
	(0.025)	(0.021)	(0.030)	(0.029)	(0.036)
*lnGI*	0.271 ***			0.440***	0.296 ***
					(0.029)
	(0.029)			(0.030)	
*lnRIS*		0.042 ***		0.411 ***	
		(0.016)		(0.041)	
*lnUIS*			−0.153***		−0.829 ***
			(0.028)		(0.095)
LnGI*lnRIS				0.233 ***	
				(0.022)	
LnGI*lnUIS					−0.463 ***
					(0.060)
Control variables	YES	YES	YES	YES	YES
Constant	3.375 ***	2.348 ***	2.542 ***	3.511 ***	3.867 ***
	(0.212)	(0.188)	(0.191)	(0.201)	(0.152)
AR(1)P	0.000	0.000	0.000	0.001	0.001
AR(2)P	0.664	0.767	0.741	0.322	0.459
Sargan (p)	0.585	0.653	0.611	0.674	0.629
Observations	480	480	480	480	480
Number of id	30	30	30	30	30

*** *p* < 0.01. Note: The values presented for AR (1), AR (2), and Sargan are the *p*-values obtained for the respective tests.

**Table 5:** Regression results for the robustness of the substituted explained variables.

Variables	Explained Variable: lnTC				
	(1)	(2)	(3)	(4)	(5)
*lnGI*	0.745 ***			0.897 ***	0.789 ***
	(0.064)			(0.067)	(0.061)
*lnRIS*		−0.005		0.502 ***	
		(0.034)		(0.087)	
*lnUIS*			−0.291 ***		−0.416 ***
			(0.050)		(0.117)
LnGI*lnRIS				0.289***	
				(0.051)	
LnGI*lnUIS					−0.050
					(0.071)
Control variables	YES	YES	YES	YES	YES
Constant	4.171 ***	1.545 ***	0.673	3.738 ***	3.342 ***
	(0.556)	(0.596)	(0.574)	(0.554)	(0.538)
Observations	540	540	540	540	540
Number of id	30	30	30	30	30
R-squared	0.691	0.607	0.632	0.711	0.725

*** *p* < 0.01.

**Table 6:** Regression results for robustness with a reduced time scale.

Variables	Explained Variable: *lnAC*				
	(1)	(2)	(3)	(4)	(5)
*lnGI*	0.543 ***			0.777 ***	0.586 ***
	(0.064)			(0.063)	(0.060)
*lnRIS*		0.105 ***		0.780 ***	
		(0.037)		(0.080)	
*lnUIS*			−0.293 ***		−0.372 ***
			(0.046)		(0.111)
LnGI*lnRIS				0.429 ***	
				(0.051)	
LnGI*lnUIS					−0.028
					(0.069)
Control variables	YES	YES	YES	YES	YES
Constant	4.963 ***	2.010 ***	1.935 ***	4.265 ***	4.529 ***
	(0.606)	(0.592)	(0.556)	(0.561)	(0.572)
Observations	480	480	480	480	480
Number of id	30	30	30	30	30
R-squared	0.642	0.591	0.618	0.705	0.685

*** *p* < 0.01.

**Table 7:** Regression results of time heterogeneity.

Variables	Explained Variable: *lnAC*			
	2003–2011		2012–2020	
	(1)	(2)	(3)	(4)
*lnGI*	0.603 ***	0.456 ***	−0.028	−0.209 **
	(0.103)	(0.096)	(0.102)	(0.102)
*lnRIS*	0.625 ***		0.208 *	
	(0.199)		(0.111)	
*lnUIS*		−0.570 **		0.090
		(0.269)		(0.160)
LnGI*lnRIS	0.416 ***		0.113 *	
	(0.102)		(0.064)	
LnGI*lnUIS		−0.113		−0.010
		(0.151)		(0.102)
Control variables	YES	YES	YES	YES
Constant	1.770 **	1.465 **	6.176 ***	5.956 ***
	(0.705)	(0.721)	(0.581)	(0.600)
Observations	270	270	270	270
Number of id	30	30	30	30
R-squared	0.759	0.751	0.256	0.253

*** *p* < 0.01, ** *p* < 0.05, * *p* < 0.1.

**Table 8:** Regression results of regional heterogeneity.

Variables	Explained Variable: *lnAC*			
	Eastern Region		Central and Western Regions	
	(1)	(2)	(3)	(4)
*lnGI*	0.854 ***	0.419 ***	0.839 ***	0.810 ***
	(0.114)	(0.124)	(0.081)	(0.068)
*lnRIS*	0.961 ***		0.195	
	(0.094)		(0.188)	
*lnUIS*		−0.690 ***		−0.138
		(0.162)		(0.162)
LnGI*lnRIS	0.533 ***		0.169	
	(0.052)		(0.127)	
LnGI*lnUIS		−0.350 ***		0.180 *
		(0.100)		(0.101)
Control variables	YES	YES	YES	YES
Constant	3.142 ***	3.239 ***	5.287 ***	5.411 ***
	(0.742)	(0.928)	(0.768)	(0.691)
Observations	198	198	342	342
Number of id	11	11	19	19
R-squared	0.736	0.616	0.708	0.760

*** *p* < 0.01, and * *p* < 0.1.

**Table 9:** Panel quantile regression results for the rationalization of the industrial structure.

Variables	Explained Variable: *lnAC*				
	(1)	(2)	(3)	(4)	(5)
	q10	q25	q50	q75	q90
*lnGI*	0.466 ***	0.424 ***	0.317 ***	0.175	−0.107
	(0.118)	(0.102)	(0.095)	(0.139)	(0.134)
*lnRIS*	1.030 ***	0.838 ***	0.828 ***	0.617 **	−0.066
	(0.256)	(0.286)	(0.198)	(0.256)	(0.259)
LnGI*lnRIS	0.555 ***	0.425 ***	0.423 ***	0.297 *	−0.145
	(0.144)	(0.155)	(0.106)	(0.152)	(0.160)
*lnQU*	−0.301 ***	−0.221 ***	−0.278 ***	−0.281 ***	−0.320 ***
	(0.044)	(0.052)	(0.052)	(0.052)	(0.062)
*lnFDI*	0.091 ***	0.025	0.009	−0.024	−0.067 **
	(0.026)	(0.021)	(0.020)	(0.032)	(0.029)
*lnHR*	−0.235	1.194 **	1.536 ***	2.220 ***	−0.069
	(0.572)	(0.573)	(0.339)	(0.697)	(0.581)
*lnER*	0.298 ***	0.311 ***	0.368 ***	0.416 ***	0.374 ***
	(0.062)	(0.038)	(0.041)	(0.032)	(0.053)
*lnUR*	2.071 ***	1.528 ***	1.709 ***	1.431 ***	2.540 ***
	(0.247)	(0.267)	(0.245)	(0.336)	(0.327)
Constant	4.917 ***	2.237 *	2.038 **	0.909	6.356 ***
	(1.137)	(1.191)	(0.815)	(1.694)	(1.298)
Number of id	30	30	30	30	30
Observations	540	540	540	540	540

*** *p* < 0.01, ** *p* < 0.05, * *p* < 0.1.

**Table 10:** Panel quantile regression results for the upgrading of the industrial structure.

Variables	Explained Variable: AC				
	(1)	(2)	(3)	(4)	(5)
	q10	q25	q50	q75	q90
*lnGI*	0.549 ***	0.397 ***	0.403 ***	0.336 ***	0.156
	(0.075)	(0.091)	(0.096)	(0.110)	(0.128)
*lnUIS*	−1.047 ***	−0.878 **	−0.991 ***	−0.369	−0.868 **
	(0.372)	(0.384)	(0.322)	(0.339)	(0.384)
LnGI*lnUIS	0.013	0.019	−0.143	0.185	−0.220
	(0.292)	(0.271)	(0.192)	(0.194)	(0.232)
*lnQU*	−0.085*	−0.131 ***	−0.179 **	−0.278 ***	−0.350 ***
	(0.044)	(0.033)	(0.069)	(0.053)	(0.068)
*lnFDI*	−0.028 *	−0.035 *	−0.035	−0.052 *	−0.089 ***
	(0.016)	(0.021)	(0.030)	(0.028)	(0.029)
*lnHR*	2.443 ***	1.990 ***	1.565 ***	1.824 ***	0.629
	(0.571)	(0.417)	(0.439)	(0.547)	(0.517)
*lnER*	0.186 ***	0.207 ***	0.292 ***	0.345 ***	0.318 ***
	(0.037)	(0.032)	(0.045)	(0.033)	(0.043)
*lnUR*	1.267 ***	1.413 ***	1.551 ***	1.733 ***	2.116 ***
	(0.233)	(0.191)	(0.313)	(0.273)	(0.273)
Constant	−0.882	0.192	1.910 *	1.862	4.559 ***
	(1.420)	(0.994)	(1.084)	(1.410)	(1.272)
Number of id	30	30	30	30	30
Observations	540	540	540	540	540

*** *p* < 0.01, ** *p* < 0.05, * *p* < 0.1.

## Data Availability

The data in this paper were collected from publicly available sources.
